# Assembly of an inexpensive rat jugular catheter button based on a split-septum needleless intravenous system

**DOI:** 10.1016/j.mex.2023.102433

**Published:** 2023-10-10

**Authors:** Mauricio Suarez, Elizabeth J. Cantrell, Ken T. Wakabayashi, Caroline E. Bass

**Affiliations:** a Neurocircuitry of Motivated Behavior Laboratory, Department of Psychology, University of Nebraska–Lincoln, 1220 T St., Lincoln, NE 68588, USA; b Rural Drug Addiction Research Center, University of Nebraska–Lincoln, 660N 12th St., Lincoln, NE 68588, USA; c Department of Pharmacology and Toxicology, Jacobs School of Medicine and Biomedical Sciences, University at Buffalo, State University of New York, Buffalo, NY 14203, USA

**Keywords:** Jugular catheter vascular access button construction, Self-administration, Jugular catheter, Cost effective, Surgery, Sterilize, In-house construction, Interlink connector

## Abstract

Rat intravenous self-administration is a widely-used animal model in the study of substance use disorders. Rats are tethered to a drug delivery system usually through a port or button that interfaces the drug delivery system with a chronic indwelling jugular vein catheter. These buttons can be purchased commercially but are costly, presenting a significant economic barrier for many researchers. Many researchers manufacture buttons in-house from a combination of individual custom made and commercially available components, resulting in large variation in terms of how the animals are handled and the longevity of catheter patency. We have developed a jugular catheter button that uses a split septum port to provide snap-on entry of a blunt cannula allowing for quick and easy attachment of the i.v. tubing. The port is constructed from commercially available split septum ports, surgical mesh and small metal cannula. The system is “needleless” which decreases the risk of infection and improves safety. The split-septum buttons are easily sterilized in-house adding to the reliability and decreases in the risk of infection. We have used this easily constructed, and inexpensive button for i.v. self-administration experiments in which 80 % of the rats maintained patency for a minimum of 35 days.

• Inexpensive method to construct a self-administration backport button.
• Utilizes inexpensive components already found in a research laboratory or commercially available.
• Can be sterilized in-house without degrading glue or components.

## Graphical abstract

**Figure:**
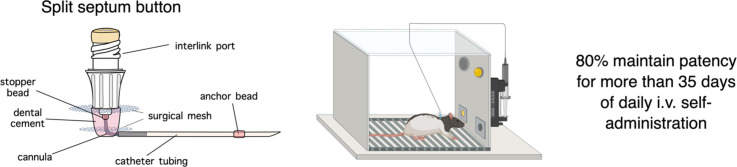

Specifications table

**Table:** 

Subject area:	Neuroscience
More specific subject area:	Drug Self-Administration
Name of your method:	Jugular catheter vascular access button construction
Name and reference of original method:	Weeks, J. R. (1962). Experimental morphine addiction: method for automatic intravenous injections in unrestrained rats. *Science*, 138(3537), 143–144.
Resource availability:	Surgical mesh, medical tubing to fit 22G cannula, catheter tubing, hypodermic tubing, tether locking cannula, Interlink septum port, dental cement, razor blade, super glue, soldering stand, small scissors.

## Introduction

Self-administration has long been considered the “gold standard” approach in modeling human drug taking behaviors in animals like rats, because of the high degree of face and construct validity compared to other experimental designs such as direct experimenter injection of the drugs. Yet, intravenous self-administration is very technically challenging. Unlike experimenter-delivered protocols, self-administration requires complex, invasive, survival surgery in order to implant the rat with a sterile chronic, indwelling catheter. The entry point of the catheter is often strategically placed between the scapulae on the rat's back so that the rat cannot reach and potentially damage the implanted catheter.

In addition to the complex implant surgery, intravenous self-administration presents numerous other challenges to the experimenter. In traditional catheter designs, the experimenter directly attaches a small diameter tube connected to the drug delivery pump to a short, small gage metal cannula exiting the catheter. The tube is most often friction-fitted to the cannula. At the end of the self-administration session, the experimenter must reverse this procedure to remove the rat from the experimental apparatus. An additional tube is friction-fitted to the metal cannula to flush the rat at the end of the procedure to help maintain catheter patency. These procedures are often challenging for less experienced experimenters as they require restraining an often excited or drugged rat, while manipulating small, difficult to see attachments. Attachment and detachment procedures need to be completed quickly with minimal stress to the rat. Thus, the experimenter needs a high degree of visual acuity and manual dexterity.

Several different commercially available ports, buttons, and adapters have been developed with distinct tethering hardware. Prices for individual sterile vascular access buttons and the associated custom-designed tether systems not only represent a significant experimental cost, but they vastly differ in experimental approach and reliability. For example, some systems employ a vest in which the rats are fitted into for each session, while others are more simply connected by magnetized buttons and tethers. The commercially available intravenous catheter systems represent a significant financial burden for many investigators, and result in vastly different approaches to handling the animals between laboratories, sometimes leading to difficulty in replicating results. Inexpensive medical components that can be assembled into a reliable and easy to use catheter button access system may increase adoption of i.v. self-administration protocols and improve standardization between studies.

In addition to cost and reproducibility between experiments, it is critical that any new catheter system remain patent and free of obstructions for the length of the experiment. Fibrin sheaths and intraluminal thrombosis are two common sources of catheter occlusion. To combat fibrins the sterility of the catheter prior to implantation is critical. All components, glues, and epoxies must not dissolve after exposure to a gas or liquid sterilant, as the heat and pressure from autoclave sterilization will destroy most catheter materials. The development of intraluminal thrombosis can be reduced through pushing a sterile anticoagulant solution regularly through the catheter (known as “flushing”). The flushing solution should be aseptic and compatible with catheter components.

Here in this protocol we demonstrate a step-by-step guide for purchasing, assembling, and testing a closed-system split septum port button. This is an inexpensive, robust, and easy to use system for laboratories interested in self-administration studies in rats.

## Method details

### Subjects

45 male Long-Evans rats were implanted with the lab made catheters described here. All procedures were reviewed and approved by the University at Buffalo Institutional Animal Care and Use Committee.

### Preparing components

A comprehensive table of components and suggested purchase locations is provided in Table 1. There are many sources for i.v. tubing, connectors, swivels, and blunt cannulae compatible with this button, many sold under the Interlink® brand. The 22 G cannula can be found from multiple sources including disassembling a 22 G needle.

**Table 1:** List of components. The name, brief description, suggested location for purchase, and helpful notes of each component needed to successfully construct the button.

Item	Description	Suggested Purchase Point	Notes
Catheter tubing	MRE 040 from BrainTree Scientific, 0.040″ OD x 0.020 ID (1.02 mm OD x 0.50 mm ID), SKU: MRE040-S2050	https://www.braintreesci.com/catheter-tubing-accessories/tubing/micro-renathane/	*Each catheter will be 9.5* *cm (female) or 11.5* *cm (male) in length*
Stopper bead tubing	Rigid tubing fitting 22 G cannula	https://www.instechlabs.com/products/tubing-connectors-pinports/tubing/co-extruded-pe-pvc	*Friction-fit PVC tubing that is friction fit to* 22 G *steel cannula/needle.*
Hypodermic Tubing	22 G, Type HTX22T (or similar)	https://componentsupplycompany.com/hypodermictubing-size-chart.php?gad=1&gclid=Cj0KCQjwuZGnBhD1ARIsACxbAVhbKXftbV337gaJjrMTW1GrzNWXXlPdLgmUwabZ9A–eUFcKhBec8YaAgvmEALw_wcB	N/A
Tether locking cannula	B. Braun Medical SafeLine® Clip Lock Cannula	https://www.medicaleshop.com/safeline-clip-lock-cannula	*Clips onto Interlink catheter to connect to drug delivery system*
Interlink septum port	2.5 cm male Interlink port (Baxter product code: 2N3379)	http://ecatalog.baxter.com/ecatalog/loadnewlookproduct.html?cid=10032&lid=10039&hid=10028&pid=1019612&categoryId=41693&subCategoryId=41703&searchAttributeHook=41695	*This URL is for the manufacturer. This product can be purchased cheaper, either sterilized or not, from several third-party distributors.*
Dental cement	Monomer and polymer	https://www.langdental.com/products/list/cat_id/5	*Powder and liquid can be found as separate items on the link provided.*
Anchor bead tubing	MRE from BrainTree Scientific, ID 0.050″−0.066″	https://www.braintreesci.com/catheter-tubing-accessories/tubing/micro-renathane/micro-renathane-065-x-030-per-ft/	*Just large enough to fit over catheter tubing.*
Small scissors	Laboratory scissors	Can be found in most labs.	N/A
Blunt Tip (for flushing)	BD Interlink™ Blunt Plastic Cannula	https://www.bd.com/en-us/products-and-solutions/products/product-page.303345#overview	*This link is for the manufacturer. This product can be purchased cheaper, either sterilized or not, from several third-party distributors.*
Soldering stand	Small stand with alligator clips used for holding microchips or small items in place.	https://www.amazon.com/Neiko-01902-Adjustable-Magnifying-Alligator/dp/B000P42O3C/ref=sr_1_5?crid=2GF9HD8DFGG&keywords=soldering%2Bstand&qid=1694655178&sprefix=soldering%2Bstand%2Caps%2C204&sr=8–5&th=1	*Also known as “helping hands”, these stands are available readily online or at hobby stores.*

**Fig. 1:**
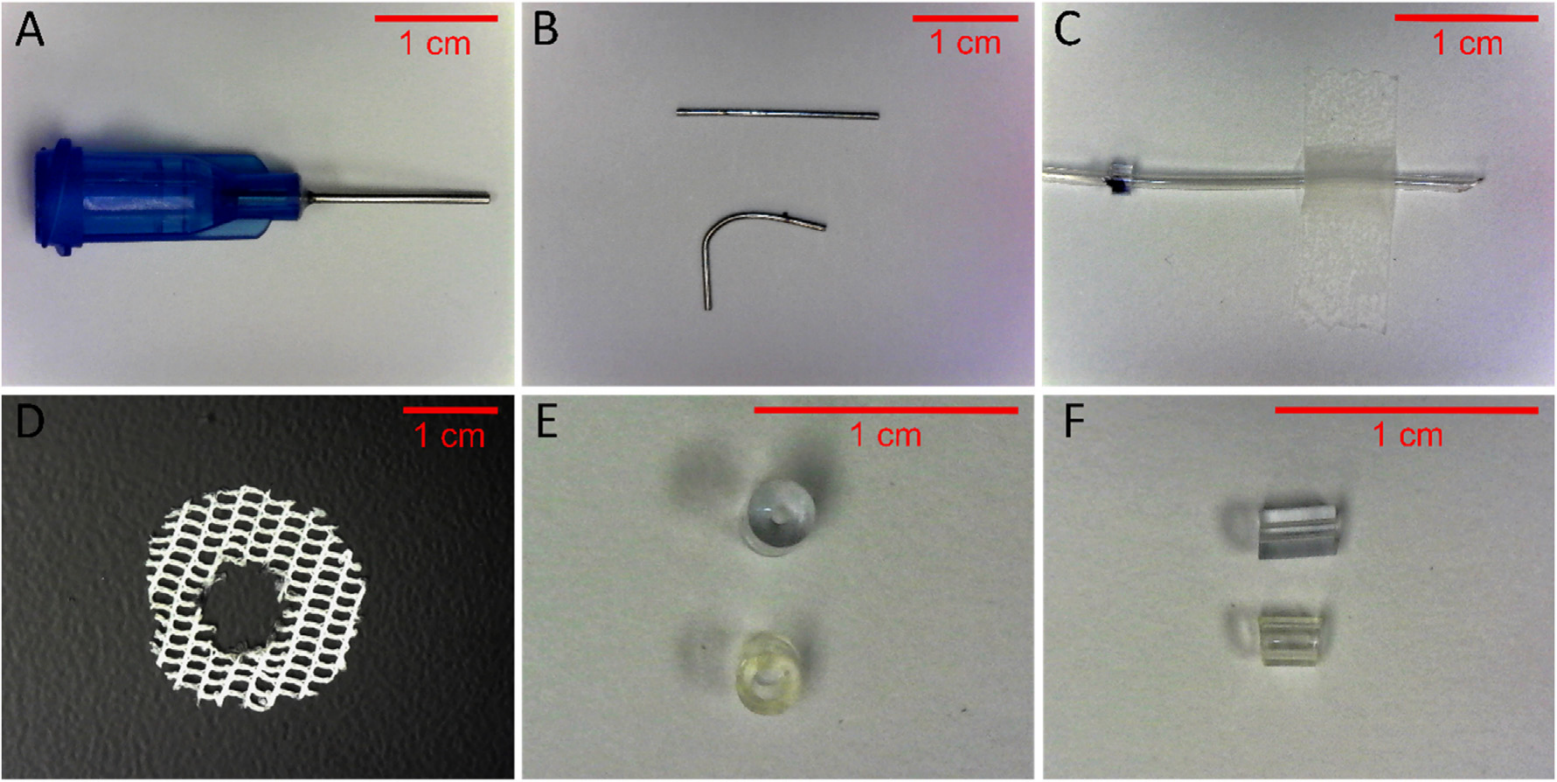
Individual components for assembly. (**A**) Blunt-tip 22 G needle that can be disassembled to obtain 22 G cannula. (**B**) A straight cannula isolated by disassembly of a blunt-tip needle or cutting hypodermic tubing to length (top). The cannula is gently bent so openings are perpendicular to each other (bottom). (**C**) The length of catheter tubing from anchor bead to beveled end, taped down to demonstrate full length. (**D**) Surgical mesh cut in a circle, with an opening that will fit it to the Interlink port and provide an anchor for the button when implanted. (**E**) Cannula stopper bead (top) and catheter anchor bead (bottom) from a top view and (**F**) side view.

22 G cannula: can be obtained from either a 22 G needle or a 22 G commercially available steel hypodermic tubing. If using a 22 G hypodermic needle (blunt-tip, Fig 1a, or a beveled needle blunted by sanding/cutting), use needle nose pliers to separate the metal tube (cannula) from the plastic needle hub. This can be done by gently pulling the metal tube out with rotation or by warming the plastic hub near the tube with a lighter flame to soften the plastic/adhesive. Use caution to ensure the cannula is not crimped in any way.

When obtaining a cannula from a 22 G separated needle, the final cannula should be trimmed to a 2-cm length and later bent at an angle (Fig 1b). The shorter end will be inserted into the Interlink split septum port and the longer end into the venous catheter. A rotary tool (e.g., Dremel®) with a Cut Off Wheel disk can be used to cut the separated needle/cannula to the appropriate length, or to trim a beveled edge if the needle is not blunt. Wear eye protection such as safety goggles when using the rotary tool. Avoid using wire snips or scissors to trim the cannula as this may produce crimps in the cannula. Crimps provide a point of attachment where biological material could accumulate.

After trimming, remove any microscopic metal debris by either sonicating the cannula or rocking for 15 min in distilled water. Briefly rinse the cannula three times with fresh distilled water and inspect them for burrs or sharp ends using a light microscope. If the end is jagged, gently smooth with a 100-grit nail file or fine sandpaper. Verify this metal cannula is straight and without holes or breaks before continuing (Fig 1b, top).

To bend the cannula, use two needle nose pliers to grip both ends of the cannula. Apply pressure to bend the cannula slowly into a curving l-shape (Fig 1b, bottom). Use caution when gripping the cannula with the pliers as applying too much pressure may crimp the ends. The final bend should gently curve, and not be at a sharp angle. The curve minimizes accumulation and adherence of biological materials to the inside of the cannula. One end of the “L” should be longer than the other. ∼0.5 cm of the cannula should be straight, followed by ∼0.5 cm comprising the bend, and the remaining ∼1 cm will form the long part of the “L” shape (Fig 1b, bottom).

Venous catheter: use a fresh, sharp razor blade to cut the MRE 040S tubing to a length of 11.5 cm for male rats, and 9.5 cm for female rats. Cut one end to a 45-degree bevel and mark 2.5 cm from the tip of the bevel with a permanent lab marker as the position of the bead anchor. The bead anchor provides a point of attachment to suture the catheter in place. This distance from the bead to the beveled tip should be sufficient for the tip of the catheter to rest well into the jugular vein (Fig 1c). The exact length should be adjusted based on the size, sex and strain of rat used. For our male Long Evans rats, the proper distance between the anchor bead and cannula tip is 2.5 cm.

Additional components: use scissors to cut two ∼23–25 mm diameter circles from Dacron® surgical mesh. Cut a ∼10 mm hole in the center of both mesh disks (Fig 1d). Create one “catheter bead” by cutting a ∼1–2 mm section of Micro-Renathane® 0.065″ x 0.030″ “bead tubing” with a razor blade. Also create one “stopper bead” by cutting a ∼3–4 mm section of “stopper bead tubing” (Fig 1e,f).

### Assembling the components

Components will be held together either with friction, cyanoacrylate glue, or dental cement.

Anchoring the catheter bead to the catheter: slide the catheter bead over the beveled end of the catheter, bringing it close to the 2.5 cm mark. A very small amount of cyanoacrylate glue should be added over the mark to affix the bead to the tube. Gently squeeze the glue allowing a drop to form at the end of the applicator that can be rubbed onto the 2.5 cm mark. Slide the catheter bead over the glue carefully with forceps or fingers, and allow to dry.

**Fig 2:**
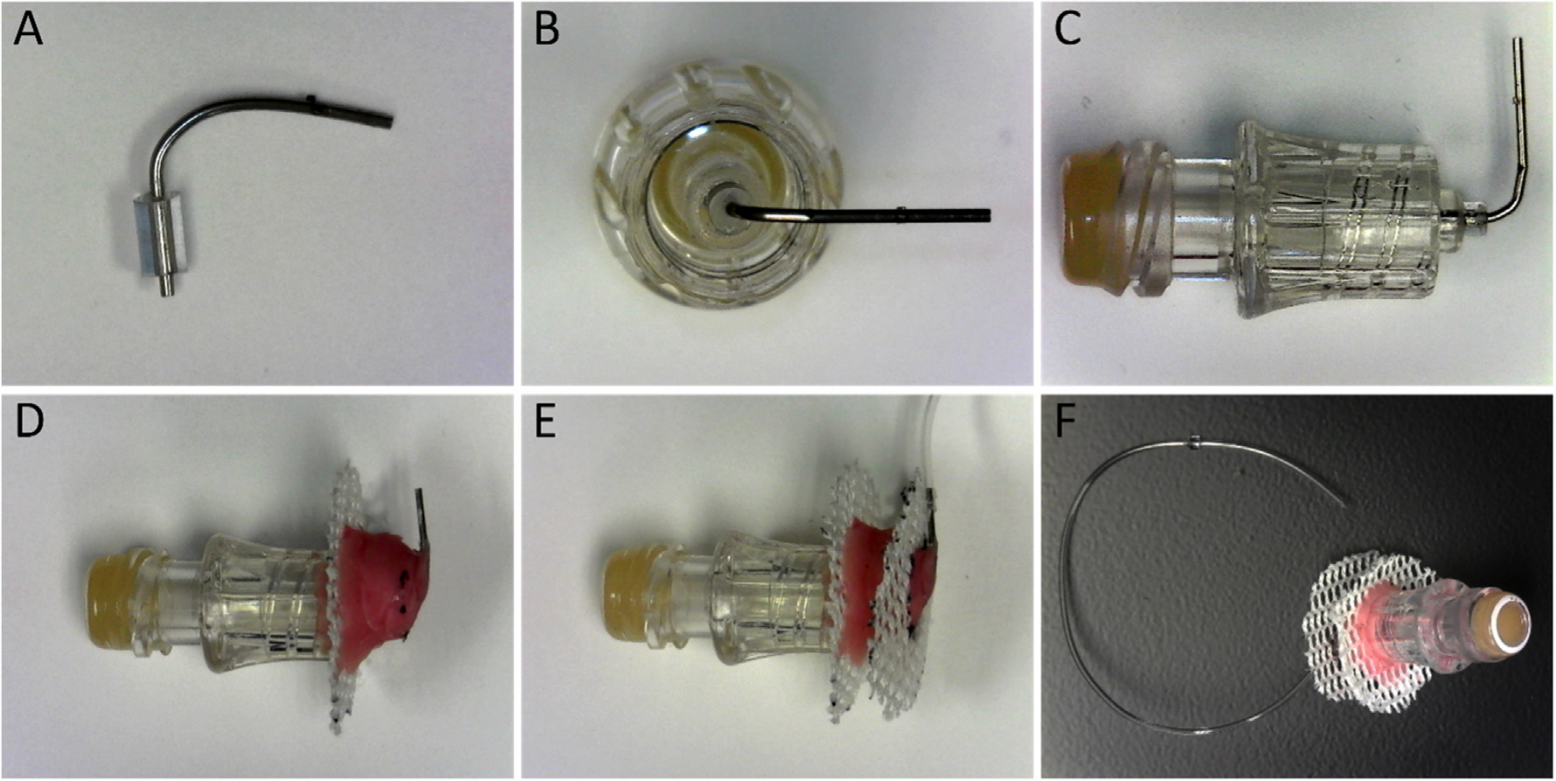
Assembled components of complete catheter button. (**A**) Bent cannula with stopper bead in place. (**B**) Top view of Interlink port once cannula and stopper bead have been secured with glue and (**C**) side view of port and cannula clearly showing the bend of the cannula is perpendicular to split septum top. (**D**) First surgical mesh disk secured to the Interlink port with dental cement and (**E**) second mesh disk secured just above the cannula and catheter. (**F**) complete catheter button assembly from a side view, ready for sterilization.

Preparing the Interlink assembly: slide the microbore “stopper” bead on the shorter end of the “L”-bent cannula and bring it close to the bend. Friction will keep this bead in place, and it will act as a “stopper” or spacer when inserted into the Interlink port (Fig 2a). Place the end of the bent cannula with the stopper bead into the open (bottom) end of the Interlink port (i.e., the end without the septum). Stand the Interlink port open-end up, clip the cannula into a soldering stand arm and lower the cannula into place. Apply a small drop of cyanoacrylate glue around the port to anchor the cannula in place and seal the port. Before continuing, confirm that the metal cannula has dried in a straight position, perpendicular to the Interlink port opening (Fig 2b,c)

Attaching the mesh disks to the Interlink assembly: the Dacron mesh disks should rest around the rim of the bottom end of the prepared Interlink assembly through the center hole of the mesh disk. Secure one Dacron mesh disk to the Interlink port assembly by placing a few drops of cyanoacrylate glue on the mesh disk along the inner rim of the Interlink port and allow it to dry. Then prepare an initial application of dental cement that is relatively fluid. Using a plastic transfer pipette, apply the first application of dental cement over the Dacron mesh around the center hole so that it flows through the mesh and contacts the base of the Interlink assembly. This provides a strong hold between the mesh and the Interlink assembly. This initial application should be relatively wide, ∼1.5 cm in diameter. Either wait or add more dental acrylic powder to the prepared dental cement so it becomes more viscous. Continue building a conical structure incorporating the bend in the metal cannula (Fig 2d). The top of the cone should be ∼0.5 cm in diameter. When cured, thread the cannula through the central hole of the second Dacron mesh disk and place this on top of the dental acrylic cone. Secure the second mesh to the dental acrylic cone with additional dental cement (Fig 2e). Allow the dental acrylic to completely cure on the assembled button assembly.

Attaching the catheter to the constructed button assembly: with forceps, slide the non-beveled end of the catheter onto the metal cannula within the button assembly. Slowly push it in as far as it will go which is generally only a few mm. Friction should be enough to hold the catheter in place. However, a small drop of cyanoacrylate glue could be added at the junction of the cannula and catheter to secure it in place. When the button is surgically implanted, both mesh discs will be sutured under the skin of the rat while the plastic portion of the Interlink port extrudes above the skin line.

A top down view of the completed catheter system can be seen in Fig 2f.

### Suggestions for catheter button use

Testing the completed catheter button for leaks: once fully cured but before sterilization, the bottom part of the catheter button should be submerged in water and air pushed through the Interlink port. To do this, attach a blunt tip plastic cannula to an empty, air-filled 1 ml syringe. Then pierce the septum with the syringe and depress the plunger to push the air through. Look for any bubbles appearing around the junction between the dental cement and any plastic component of the Interlink tube, as well as where the catheter meets the cannula.

In-house sterilization: catheter buttons can safely be cold sterilized using a 2.65 % glutaraldehyde solution, such as commercially available Wavicide®. To sterilize, follow the manufacturer's guidelines for sterilizing polyurethane-based tubing. Once sterilized, use sterile forceps and gloves to first remove the button from the glutaraldehyde solution, then fill the length of the button and catheter with 70 % ethanol using a 1 ml syringe; filling the length disinfects the inner lumen of the catheter. Soak the ethanol filled catheter button in a 70 % ethanol solution for at least 8 h. If catheters are not to be implanted within the week, they should be stored in a cool, dry environment.

Catheter implantation and tethering: immediately before implantation, flush the catheter with sterile saline to hydrate and remove excess ethanol. The surgical procedures for implanting a jugular catheter are beyond the scope of this paper. However, guides on jugular catheter surgery and tethering animals can be found elsewhere [[1], [2]]. These Interlink button catheters are designed to easily clip on to a B. Braun Medical SafeLine® Clip Lock Cannula tether system. These clip locks can be attached to stainless steel spring tethers containing tubing connected to drug dispensing apparatus common in self-administration set ups. There are several options for injectors, connectors, tubing and swivels that can be used to safely flush animals and connect to these drug dispensing systems.

### Maintaining catheter patency

Parameters for determining patency: 45 male Long-Evans rats were used to determine catheter patency. These rats were part of cocaine self-administration experiments and their patency was determined with two methods. The first method was to periodically administer a low dose of ketamine HCl (10 mg/ml, prepared in sterile 0.9 % saline, USP, ∼0.1 ml per test) manually through the catheter every ∼7 days to determine if this infusion would induce an apparent loss of motor coordination in rats. This effect usually occurs within 10 s after ketamine administration. Second, the rats were consistently monitored during their self-administration sessions for appropriate discrimination between active and nonactive levers. Pressing the active lever delivered a reinforcing dose of cocaine (0.5 mg/kg/infusion), while pressing the inactive lever during these sessions had no programmed consequences. The appropriate criterion for discrimination was ∼5:1 drug-paired lever presses to nondrug-paired lever presses.

The self-administration experiments were conducted daily for ∼3 h a day for an average of 35 days. 36 of 45 subjects (80 %) completed all of their self-administration sessions while 9 or 20 % were removed due to loss of patency. The catheter remained patent for as long as 40 days in some rats, which was the longest time point tested.

## Conclusions

Here we demonstrate an easily assembled, robust, and long-lasting self-administration catheter button constructed from inexpensive medical components. The use of dental cement allows for cold sterilization without the risk of the adhesive dissolving, bypassing the need for expensive gas sterilizers or autoclaves. Because the vascular system of the rat is accessed through a split septum, there is no need for cannula dummies or screwcaps. The Interlink system is well vetted in humans and in veterinary practices resulting in low infection rates. The ease of piercing the split septum allows us to quickly train lab personnel of all levels to flush and tether the rats to the self-administration system. The quick connection results in less restraint and stress on the rat. We conclude that this method generates inexpensive and reliable catheter buttons that a lab can easily produce, and which is a much cheaper alternative to commercially available proprietary buttons.

## Ethics statements

All procedures and experiments complied with the ARRIVE guidelines and the National Institutes of Health guide for the care and use of laboratory animals (NIH Publications No. 8023, revised 1978). All procedures and experiments were approved by the Institutional Animal Care and Use Committee at the University at Buffalo.

## CRediT authorship contribution statement

**Mauricio Suarez:** Conceptualization, Methodology, Visualization, Investigation, Writing – original draft, Writing – review & editing. **Elizabeth J. Cantrell:** Writing – review & editing. **Ken T. Wakabayashi:** Writing – review & editing. **Caroline E. Bass:** Conceptualization, Writing – review & editing, Supervision, Resources, Funding acquisition, Project administration.

## Data Availability

Data will be made available on request.
